# Membrane Cholesterol Regulates Lysosome-Plasma Membrane Fusion Events and Modulates *Trypanosoma cruzi* Invasion of Host Cells

**DOI:** 10.1371/journal.pntd.0001583

**Published:** 2012-03-27

**Authors:** Bárbara Hissa, Jacqueline G. Duarte, Ludmila F. Kelles, Fabio P. Santos, Helen L. del Puerto, Pedro H. Gazzinelli-Guimarães, Ana M. de Paula, Ubirajara Agero, Oscar N. Mesquita, Cristina Guatimosim, Egler Chiari, Luciana O. Andrade

**Affiliations:** 1 Department of Morphology, Federal University of Minas Gerais, Belo Horizonte, Brazil; 2 Department of Physics, Federal University of Minas Gerais, Belo Horizonte, Brazil; 3 Department of General Pathology, Federal University of Minas Gerais, Belo Horizonte, Brazil; 4 Department of Parasitology, Federal University of Minas Gerais, Belo Horizonte, Brazil; Universidad Autónoma de Yucatán, Mexico

## Abstract

**Background:**

Trypomastigotes of Trypanosoma cruzi are able to invade several types of non-phagocytic cells through a lysosomal dependent mechanism. It has been shown that, during invasion, parasites trigger host cell lysosome exocytosis, which initially occurs at the parasite-host contact site. Acid sphingomyelinase released from lysosomes then induces endocytosis and parasite internalization. Lysosomes continue to fuse with the newly formed parasitophorous vacuole until the parasite is completely enclosed by lysosomal membrane, a process indispensable for a stable infection. Previous work has shown that host membrane cholesterol is also important for the T. cruzi invasion process in both professional (macrophages) and non-professional (epithelial) phagocytic cells. However, the mechanism by which cholesterol-enriched microdomains participate in this process has remained unclear.

**Methodology/Principal Finding:**

In the present work we show that cardiomyocytes treated with MβCD, a drug able to sequester cholesterol from cell membranes, leads to a 50% reduction in invasion by *T. cruzi* trypomastigotes, as well as a decrease in the number of recently internalized parasites co-localizing with lysosomal markers. Cholesterol depletion from host membranes was accompanied by a decrease in the labeling of host membrane lipid rafts, as well as excessive lysosome exocytic events during the earlier stages of treatment. Precocious lysosomal exocytosis in MβCD treated cells led to a change in lysosomal distribution, with a reduction in the number of these organelles at the cell periphery, and probably compromises the intracellular pool of lysosomes necessary for *T. cruzi* invasion.

**Conclusion/Significance:**

Based on these results, we propose that cholesterol depletion leads to unregulated exocytic events, reducing lysosome availability at the cell cortex and consequently compromise *T. cruzi* entry into host cells. The results also suggest that two different pools of lysosomes are available in the cell and that cholesterol depletion may modulate the fusion of pre-docked lysosomes at the cell cortex.

## Author Summary

*Trypanosoma cruzi*, is the etiological agent of a neglected tropical malady known as Chagas' disease, which affects about 8 million people in Latin America. 30–40% of affected individuals develop a symptomatic chronic infection, with cardiomyopathy being the most prevalent condition. *T. cruzi* utilizes an interesting strategy for entering cells: *T. cruzi* enhances intracellular calcium levels, which in turn trigger the exocytosis of lysosomal contents. Lysosomes then donate their membrane for the formation of the parasitophorous vacuole. Membrane rafts, cholesterol-enriched microdomains in the host cell plasma membrane, have also been implicated in *T. cruzi* invasion process. Since both plasma membrane and lysosomes collaborate in parasite invasion, we decided to study the importance of these membrane domains for lysosomal recruitment and fusion during *T. cruzi* invasion into host cells. Our results show that drug dependent depletion of plasma membrane cholesterol changes raft organization and induces excessive lysosome exocytosis in the earlier stages of treatment, leading to a depletion of lysosomes near the cell cortex, which in turn compromises *T. cruzi* invasion. Based on these results, we propose that cholesterol depletion leads to unregulated exocytic events of pre-docked lysosomes, reducing lysosome availability at the cell cortex and consequently compromising *T. cruzi* infection.

## Introduction

*Trypanosoma cruzi*, the etiological agent of Chagas' disease, is a protozoan parasite capable of invading several types of non-professional phagocytic cells including fibroblasts, endothelial cells, and myocytes [1], [2]. Invasion occurs when parasite attaches to and stimulates host cell, leading to intracellular calcium signaling events that culminate with lysosome recruitment and fusion with the host cell plasma membrane and formation of the parasitophorous vacuole [3], [4], [5]. Several factors, such as parasite membrane proteins and proteins shed or secreted by the parasite, are known to interact with host cell membrane receptors during the *T. cruzi* entry process into host cells [6], [7], [8], [9], [10], [11]. Therefore, host cell plasma membrane plays an important role in *T. cruzi* adhesion and internalization, and modulates intracellular signaling events that are imperative for a successful infection of host cells by the parasite.

The host cell plasma membrane is a complex structure formed by a fluid and dynamic lipid bilayer to which various proteins and ligands with different biological functions are associated [12]. It is well established that the plasma membrane is not a homogeneous structure. On the contrary, the plasma membrane not only presents an asymmetric lipid distribution over its exoplasmic and cytoplasmic leaflets [13], but also shows inhomogeneities in the lateral distribution of lipids. In 1997, these lateral asymmetries were well described by Simons and Ikonen as sphingolipids and cholesterol-enriched microdomains known as lipid rafts [14].These microdomains are likely to be kept together due to lateral association between carbohydrate heads of glycosphingolipids and the presence of cholesterol molecules filling the empty area between those lipids. Several proteins were also identified inside lipid rafts: e.g., GPI- anchored proteins, transmembrane proteins, and tyrosin kinases among others [15], [16], [17]. Due to their specific characteristics, lipid rafts play several roles in cell signaling, molecular organization and membrane trafficking [18]. Beyond these cellular functions, several works show that these microdomains are also involved in internalization of pathogens like virus, bacteria and protozoans [19], [20], [21]. Recently, two independent groups have shown that cholesterol-enriched regions might be involved in *T. cruzi* entry into host cells [22], [23]. According to these authors, cholesterol localized in cell membranes contributes significantly to the infectivity of metacyclic trypomastigotes and extracellular amastigotes in non-professional phagocytic cells (Vero and HeLa cells [23]), or to the infectivity of tissue culture trypomastigotes in professional phagocytic cells [22]. In both works, methyl-beta cyclodextrin (MβCD) depletion of host cell membrane cholesterol considerably reduced parasite infectivity. However, the mechanism by which cholesterol-enriched membrane microdomains contribute to infectivity of *T. cruzi* was not elucidated.

It is well established that several proteins and receptors associated with lipid rafts are responsible for triggering intracellular signaling cascades [24]. *T. cruzi* interaction with host cells also evokes various host signaling events that culminate with recruitment and fusion of lysosomes with the plasma membrane and the subsequent formation of a viable parasitophorous vacuole, without which parasites are able to escape from its host cell [3], [4], [5], [25]. SNARE complex proteins (*Soluble N-ethylmaleimide-sensitive factor Attachment protein Receptor*), SNAP-23 (Synaptosome-Associated Proteins) and Syntaxin 4 at the plasma membrane and VAMP-7 on lysosomal membranes, have been shown to coordinate lysosome fusion with plasma membrane [26]. Interestingly, it has been demonstrated that SNARE protein complexes are preferentially localized in lipid rafts [27], [28], [29].

Since lysosomal fusion is essential for the successful invasion of *T. cruzi* into host cells and signaling proteins, as well as proteins of the SNARE complex, reside in rafts, we decided to evaluate if altering the concentration and distribution of cholesterol interferes with the *T. cruzi* invasion process and if these changes affected the lysosomal fusion events during this process. We tested this by sequestering the cholesterol from cell membranes of primary mouse cardiomyocytes with MβCD before exposure to *T. cruzi* trypomastigotes. Our results show that the diminishment of *T. cruzi* entry into host cells after cholesterol sequestration is a consequence of the reduction in lysosomal recruitment during the formation of the parasitophorous vacuole. We then demonstrate that this decrease in lysosome recruitment and fusion during parasite entry into host cells is a consequence of unregulated lysosomal exocytosis events, which reduce the number of lysosomal vesicles that are normally localized near the cell cortex and available for the formation of the parasitophorous vacuole.

## Methods

### Ethics statement

All animals were maintained in our animal facilities in compliance with the guidelines of the UFMG (Universidade Federal de Minas Gerais) ethics committee for the use of laboratory animals (protocol 45/2009 approved by CETEA-UFMG) and are in accordance with CONCEA, the Brazilian institution that regulates animal husbandry.

### Cells and parasites

Primary cultures of murine neonatal cardiomyocytes were prepared from fifteen neonatal (1–3 day old) Swiss mice. After euthanization by decapitation hearts were removed aseptically and kept on ice in Hanks' balanced salt solution (HBSS) (Sigma-Aldrich) (pH 7.4). Hearts were washed three times with fresh ice-cold HBSS, minced into small fragments and washed twice during mincing. Cardiac tissue was first dissociated overnight at 4°C in an enzymatic solution containing 0.05% (vol/vol) trypsin-EDTA 0.25% (Sigma-Aldrich) in HBSS and then trypsin was inhibited with 1 mL of soybean trypsin inhibitor (Sigma-Aldrich), 1 mg/mL in HBSS. Next, samples were submitted to a second dissociation step with 5 mL collagenase type 2 (Worthington), 1 mg/mL in Leibovitz medium (Sigma-Aldrich). The cell suspension was filtered through a 70 µm cell strainer and then centrifuged at 300 g for 5 minutes. The pellet containing dissociated cells was resuspended in high-glucose DMEM (Invitrogen), supplemented with 10% (vol/vol) fetal bovine serum (FBS) and 1% (vol/vol) penicillin/streptomycin (100 U/mL/100 µg/mL) (Invitrogen). The cell suspension was pre-plated for 2 hours at 37°C in a 5% CO_2_ incubator in order to remove most fibroblasts and other non-muscle cells. Supernatant enriched in cardiomyocytes was then collected and seeded at a density of 1,0×10^5^ cells/well onto 24-well plates containing round coverslips pre-treated with fibronectin (Sigma-Aldrich). Cells were incubated at 37°C in a humidified atmosphere containing 5% CO_2_ for 72 hours before experimental procedures. New cultures were prepared for each experiment.

Tissue culture trypomastigotes from *T. cruzi* (*T. cruzi* TCTs), Y strain, were obtained from the supernatant of infected monolayers of the LLC-MK2 cell line and purified as described previously [30].

### Cell treatment

For cholesterol depletion from host cell plasma membrane, cardiomyocytes were washed three times with phosphate buffered saline containing Ca^2+^ and Mg^2+^ (PBS+/+) and incubated in high-glucose DMEM containing different concentrations of methyl-beta cyclodextrin (MβCD) (Sigma-Aldrich) for 45 minutes at 37°C. Alternatively, cells were incubated in high-glucose DMEM containing different concentrations of hydroxypropyl-gamma cyclodextrin (HγCD) (Sigma-Aldrich), an inactive analog of MβCD which does not release cholesterol from cells in significant amounts as an internal control. After drug treatment, monolayers were washed three times with PBS+/+ and used in the different experimental procedures.

Cholesterol repletion was performed by incubating cells, previously treated with the highest concentration of MβCD, in high-glucose DMEM containing 0.05 mM of water soluble cholesterol (WSC) (Sigma-Aldrich), for 30 minutes at 37°C.

### Cell invasion assays

Cardiomyocyte cultures pre-treated or not with MβCD were exposed to purified *T. cruzi* TCTs ressuspended in high-glucose DMEM at a multiplicity of infection (MOI) of 50. The infection was performed for 40 minutes at 37°C. After infection, monolayers were washed at least four times with PBS+/+ and fixed in 4% (wt/vol) paraformaldehyde (Sigma-Aldrich)/PBS+/+. After fixation, cells were processed for immunofluorescence or other labeling.

### Immunocytochemistry

After treatment, infection and fixation, coverslips containing attached cells were washed with PBS+/+, incubated for 20 min with PBS+/+ containing 2% bovine serum albumin (BSA) (Sigma-Aldrich) and processed for an inside/outside immunofluorescence invasion assay as described previously [30]. Briefly, cells were fixed and extracellular parasites were immunostained using a 1∶500 dilution of rabbit anti-*T. cruzi* polyclonal antibodies in PBS containing 2% BSA (PBS/BSA) followed by labeling with Alexa Fluor-546 conjugated anti-rabbit IgG antibody (Invitrogen).

After extracellular parasite staining, cells were permeabilized using a solution containing 2% BSA and 0.5% saponin (Sigma-Aldrich) in PBS (PBS/BSA/saponin) for 20 minutes. Host cell lysosomes were then immunostained using a 1∶50 dilution of rat anti-mouse LAMP-1 hybridoma supernatant (1D4B; Developmental Studies Hybridoma Bank, USA) in PBS/BSA/saponin for 45 minutes followed by labeling with Alexa Fluor-488 conjugated anti-rat IgG antibody (Invitrogen), as described previously [3]. Subsequently, the DNA of both host cells and parasites were stained for 1 min with 10 µM of DAPI (Sigma-Aldrich), mounted with ProLong Gold antifade reagent (Molecular Probes), and examined on a Zeiss Axioplan microscope equipped with an oil immersion objective (100×, 1,3 NA) and with an Axiocam HRC camera controlled by Axiovision Software (Zeiss).

Cell surface area for the different conditions (treated or not with MβCD or HγCD) was determined using a plasma membrane labeling agent, CellMask orange plasma membrane stain (Invitrogen), according to manufacturer instructions. Briefly, control non-treated cardiomyocytes, as well as cardiomyocytes treated with either 15 mM MβCD, 15 mM HγCD or 15 mM MβCD followed by incubation with 0.05 mM of WSC, were washed with PBS+/+ and incubated with a solution of 5 µg/mL CellMask in DMEM without serum for 5 minutes, at 37°C. After this period, cells were fixed in a solution of 4% paraformaldehyde in fresh media for 10 minutes, at 37°C. Coverslips were then washed three times with PBS+/+ and mounted using antifade medium. Images were collected immediately afterwards using an Olympus FV300 confocal/WX61WI microscope system (Figure S1).

### Filipin and GM1 labeling

After cholesterol depletion and repletion, cells were fixed and labeled with either Filipin III (Sigma-Aldrich) or subunit B of cholera toxin-Alexa Fluor 488 (CTXb) (Sigma-Aldrich) for detection of plasma membrane cholesterol and GM1 ganglioside, respectively, as described previously [23]. Briefly, after cholesterol depletion/replenishment cells were washed and fixed with paraformaldehyde as described above. After fixation, cells were permeabilized with PGN solution (PBS+/+, 0.15% gelatin and 0.1% sodium azide) containing 0.1% saponin for 15 minutes. Following permeabilization, cells were labeled with CTXb (1 µg/mL) diluted in PGN for 30 minutes. Cells labeled with CTXb were analyzed using the confocal microscope system described above. Images were collected and analyzed using Fluoview version 5.0. In order to visualize the distribution of cholesterol in cell plasma membrane, fixed cells were labeled with both Filipin III (for cholesterol detection) (100 µg/mL in PGN) and DAPI (for nuclei staining) and examined on a Zeiss Axioplan microscope equipped with an Axiocam HRC camera controlled by Axiovision Software (Zeiss). For quantitative assays of cholesterol depletion/repletion, only Filipin III was stained. Images of 10 fields/coverslip were collected with an oil immersion objective (100×, 1,3 NA), using the same CCD exposure time and illumination intensity and then analyzed using the ImageJ image processing program (http://rsb.info.nih.gov/ij/) for fluorescence quantification. Four equal squared areas were chosen in each image and the fluorescence intensity of each area was determined. These values were then used to calculate the average fluorescence of each image and then of each experimental group.

### β-hexosaminidase secretion assay

To evaluate the level of lysosomal exocytosis after treatment with MβCD, a time dependent β-hexosaminidase secretion assay was performed according to previous work [31]. Briefly cells were exposed to 10 mM MβCD or HγCD for different incubation periods in the presence or absence of calcium. In the latter calcium was substituted by the same concentration of Mg^2+^ and EGTA was also added. After drug incubation, 350 µL of extracellular media was collected and adhered cells were lysed using 1% Triton x-100 (Sigma-Aldrich) in PBS. Extracellular media and lysates were incubated with 50 µL of β-hexosaminidase substrate, 6 mM 4-methylumbelliferyl-N-acetyl-B-D-glucosaminide (Sigma-Aldrich), dissolved in Na-citrate-PO_4_ buffer (pH 4.5). Reactions were stopped by adding 100 µL of Stop Solution (2 M Na_2_CO_3_-H_2_O, 1.1 M glycin) and read at excitation 365 nm and emission 450 nm in a spectrofluorimeter (Synergy 2, Biotek in the Center of Flow Cytometry and Fluorimetry, Department of Biochemistry and Immunology, ICB-UFMG).

### Cell viability assay

After incubation with either 10 mM MβCD or HγCD for 45 minutes, or with 10 µM Ionomycin for 10 minutes, in the presence or absence of calcium, cardiomyocytes were trypsinized, pelleted and incubated with Hypotonic Fluorochrome Solution (HFS - 50 µg/mL Propidium Iodide (PI) in 0.1% sodium citrate) for 4 hours at 4°C protected from light. This assay was performed in order to quantify cell death after drug treatment according to previous work [32]. The PI fluorescence of 20,000 individual cells was measured using a Becton Dickinson FACscan (BD Biosciences, USA) and data were analyzed using the Cell Quest Pro software (BD Biosciences, USA).

### Lysosomal dispersion analysis

Using a set of images, obtained from treated and non-treated cells with labeled nuclei and lysosomes, we analyzed the mean lysosome distance relative to the mean center position in the respective nuclei. For each image (associated with a specific drug treatment), we visually selected a number of isolated nuclei, and using its borders' positions (i.e., the image's [X,Y] coordinates), we computed the mean center position and the mean radius (namely R) of each nucleus.

A computational code has been written using the IDL (Interactive Data Language) programming language in order to assist in these calculations. Subsequently, using the (X,Y) position of each lysosome distributed around each nucleus, the distances to the center were calculated. An average distance (namely D) was computed by using the distances of each lysosome relative to the center. Lysosomes farther than 2 radii from cell center were excluded from the computation of this average value. Finally, the mean lysosome distance (D) relative to the mean nucleus' radius (R) was defined as the ratio D/R. This procedure was repeated for the maximum number of isolated cells available from the image sets of each drug treatment. The results of this analysis are a distribution of D/R values associated to each drug treatment, and are represented as histograms.

### Statistical analysis

All experiments were performed in triplicate and repeated at least three times. For Filipin labeling, one-way ANOVA followed by post- hoc comparison Newman-Keuls was performed to evaluate statistically significant differences. For invasion assays, a minimum of 200 cells was counted per coverslip and analyzed using the Student's t-test. For histogram distributions, the cumulative frequency was calculated and analyzed using the Kolmogorov-Smirnov statistical test.

## Results

### MβCD treatment does reduce the amount of membrane bound cholesterol

**Figure 1:**
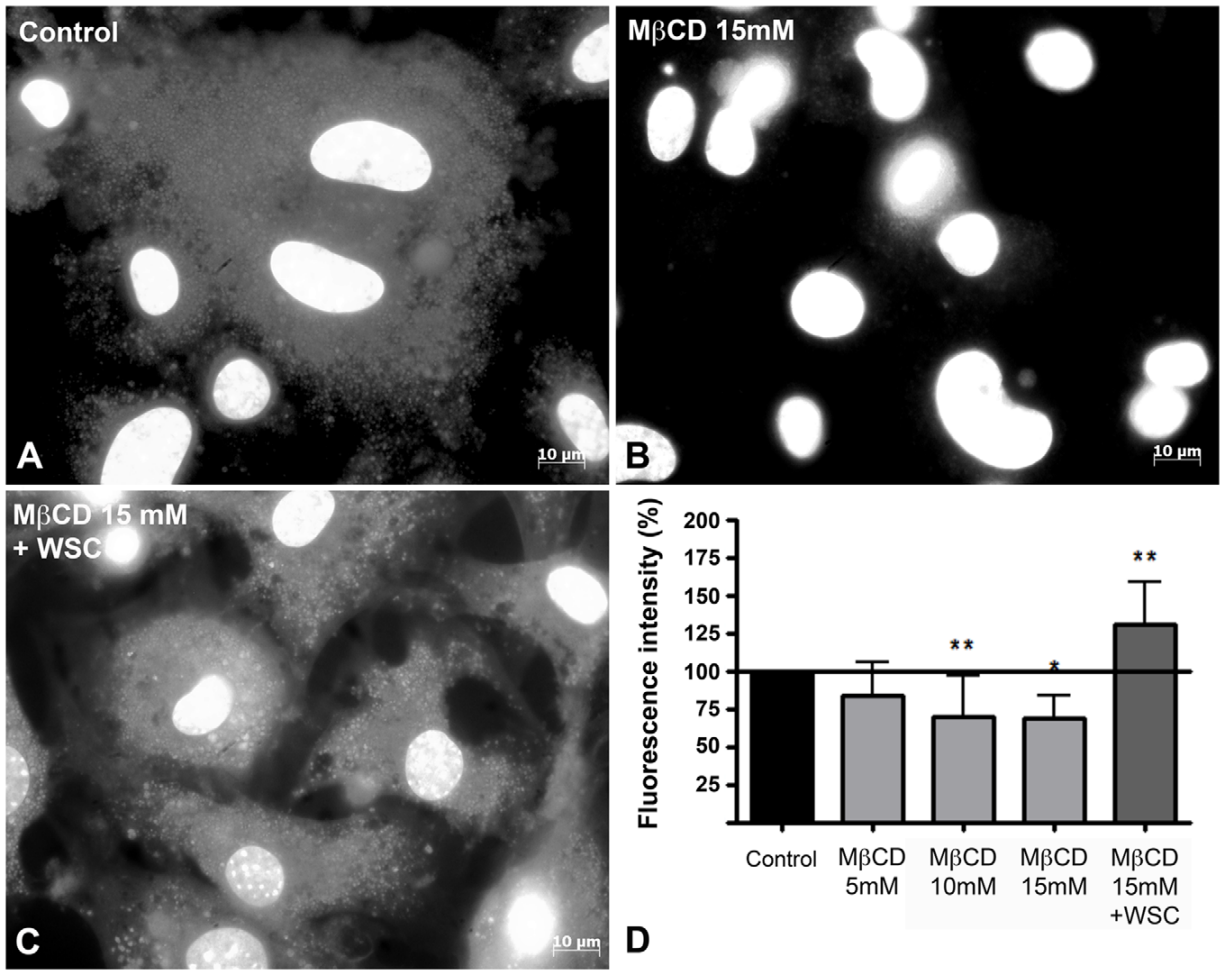
MβCD treatment is effective in sequestering cholesterol from the plasma membrane. (A) Control, (B) 15 mM MβCD treated and (C) 15 mM MβCD followed by 0.05 mM WSC treated cardiomyocytes show significant changes in Filipin labeling. Cholesterol-depleted cells (B) reveal very little Filipin labeling, whereas cholesterol-replenished cells show strong labeling for cholesterol, similar to control cells. (D) Cardiomyocytes treated with 5, 10 or 15 mM of MβCD reveal a substantial decrease in Filipin labeling in a dose-dependent manner whereas cholesterol replenishment with 0.05 mM of WSC returns Filipin fluorescence to control values. Normalized data are shown as mean of triplicates ±SD. Asteriks indicate statistically significant differences (* p < 0.05 and ** p < 0.01, One way ANOVA followed by Newman-Keuls) between control and treated cells. Scale bar: 10 µm.

Before investigating the influence of cholesterol removal in invasion of cardiomyocytes by *T. cruzi* TCTs, we tested whether MβCD treatment was able to sequester cholesterol from cultured murine cardiomyocyte membranes. Cells treated with different concentrations of MβCD were stained with Filipin III, a sterol-binding fluorescent polyene, which is able to bind to cholesterol present in the plasma membrane [33]). Fluorescence microscopy images of cells pre-treated with 15 mM of MβCD (Fig. 1B) show decreased staining with Filipin when compared to control cells (Fig. 1A), confirming the removal of cholesterol upon drug treatment. Addtionally, if MβCD treated cultures were incubated with 0.05 mM of water soluble cholesterol (WSC) diluted in serum free media, causing membrane cholesterol to be replenished, surface staining with Filipin was recovered (Fig. 1C). Quantitative assays, using the ImageJ program to quantify fluorescence intensity, were also performed to measure cell staining with Filipin III before and after treatment with MβCD, as well as upon cholesterol depletion and repletion. As was seen for the qualitative assays, Filipin staining of cholesterol decreased upon treatment with increasing concentrations of MβCD (Fig. 1D). A small reduction was observed upon treatment with 5 mM of the drug, increasing to 30% after treatment with a concentration of 15 mM. Cholesterol replenishment with WSC reverted the process, showing an increase in cell staining as a consequence of the increase in membrane bound cholesterol.

### Cholesterol depletion diminishes *T. cruzi* cell invasion into primary neonatal murine cardiomyocytes

Previous studies have shown that host cell cholesterol-enriched microdomains play a significant role in the adhesion and internalization of *T. cruzi* TCTs in professional phagocytic cells [22], as well as *T. cruzi* metacyclic trypomastigotes or extracellular amastigotes forms in non-professional phagocytic cells [23]. Since *T. cruzi* TCT invasion into non-professional phagocytic cells, such as cardiomyocytes, is an important event during clinical infection, in the present work we first investigated whether host cell cholesterol was also relevant in cell invasion of this *T. cruzi* form into cultured, primary neonatal murine cardiomyocytes.

**Figure 2:**
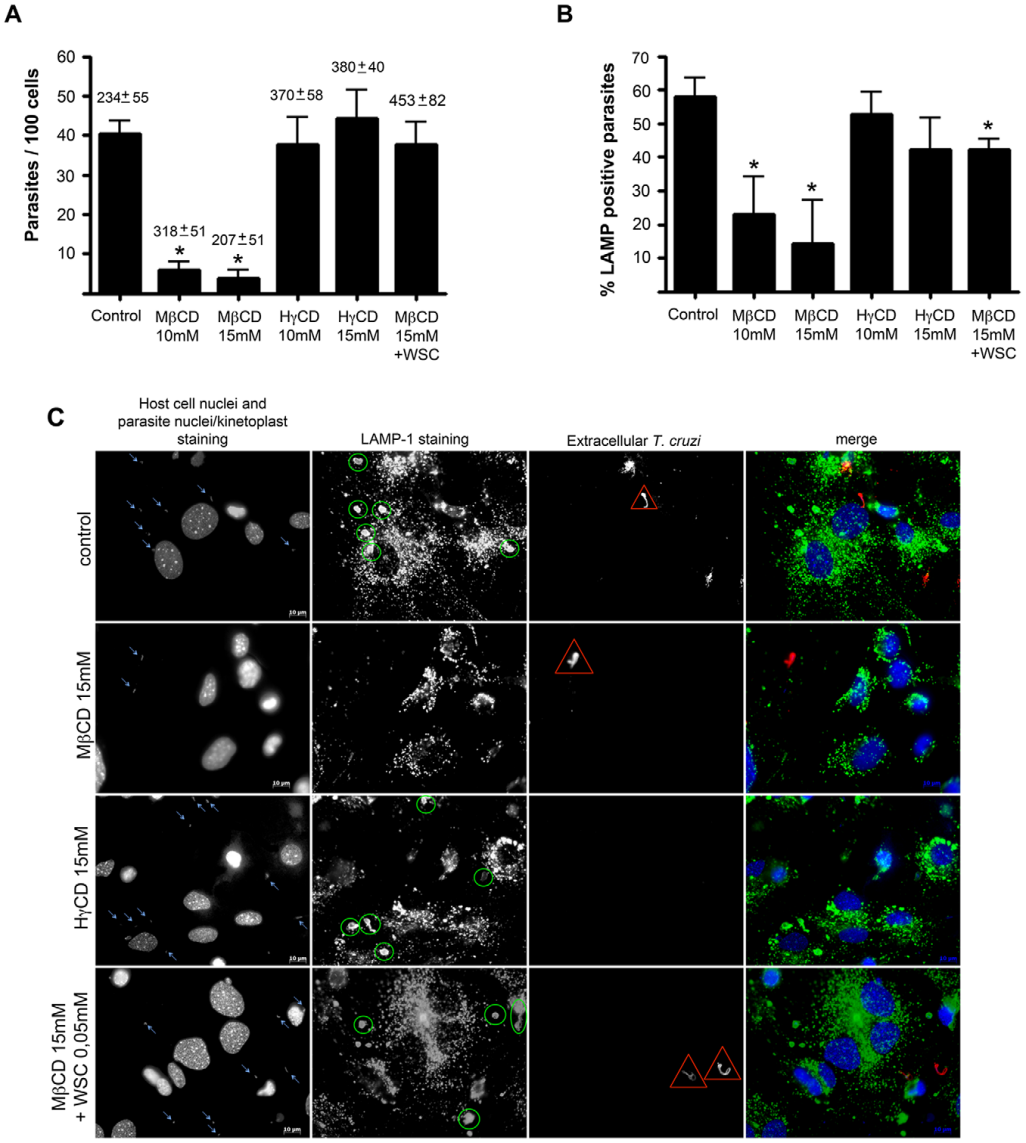
*T. cruzi* invasion of cells and association with LAMP-1 in cardiomyocytes decreases after cholesterol depletion. Cardiomyocytes pre-treated or not with different cyclodextrins were washed and challenged with *T. cruzi* trypomastigotes at a M.O.I of 50, for 40 minutes at 37°C, then fixed and processed for immunofluorescence detection of total intracellular parasites, as well as intracellular parasites associated with LAMP-1 (a lysosomal marker). Both *T. cruzi* internalization (A) and association with host LAMP-1 (B) diminishes after incubation with 10 and 15 mM of MβCD but not after treatment with 10 and 15 mM of HγCD. Cholesterol replenishment after treatment with 15 mM MβCD reverts the effect of the drug on parasite cell invasion (A), and at least partially on LAMP-1 association (B). The average number of cardiomyocytes ±SD per 10 counted fields in each coverslip is shown above the bars (A). Data are shown as mean of triplicates ±SD. Asteriks indicate statistically significant differences (p < 0.05, Student's t test) between control and treated cells. (C) Representative panels of *T. cruzi* invasion and association with host cell lysosomes, revealed by immunocytochemistry. Total cell and parasite nuclei, as well as parasite kinteoplast DNA were labeled with DAPI; lysosomes were labeled with anti-LAMP 1 antibody followed by secondary IgG labeled with Alexa Fluor 488; extracellular parasites in the field were labeled with anti-*T.cruzi* antibody followed by secondary IgG labeled with Alexa Fluor 546. From top to bottom: control cells, 15 mM MβCD treated cells, 15 mM HγCD treated cells and 15 mM MβCD treated cells followed by incubation with 0.05 mM of WSC. Blue arrows show total *T. cruzi* trypomastigotes in the field, yellow ellipsoids show lysosomal associated trypomastigotes, red triangles points out extracellular trypomastigotes and the last column shows the merge of the three previous. Scale bar: 10 µm.

Previously plated cells were incubated with 10 or 15 mM of MβCD for 45 minutes at 37°C (i.e., conditions known to be effective in membrane cholesterol removal [31]), followed by a 40 minute exposure to *T. cruzi* TCTs. As observed before for macrophages [22], we determined that cholesterol depletion from murine cardiomyocytes membranes also leads to a reduction in *T. cruzi* TCT invasion (Fig. 2A and C). Upon treatment with 10 mM or 15 mM MβCD, a reduction of 85–90% in *T. cruzi* cell invasion was observed (Fig. 2A). As a control for MβCD treatment, cells were treated with the same concentrations of HγCD, a cyclodextrin analog of MβCD with low affinity for cholesterol. HγCD-treated cells did not show any differences in *T. cruzi* cell invasion as compared to control non-treated cells (Fig. 2A and C). Plus, reduction in the number of invading *T. cruzi* TCTs after MβCD treatment did not appear to be the result of host cell death upon drug treatment since the total number of cardiomyocytes per 10 fields in all conditions tested was not statistically different from each other (Fig. 2A, number above bars).

### *T. cruzi* cell invasion is re-established after membrane cholesterol replenishment

To address whether the reduced *T. cruzi* invasion observed after host cell treatment with MβCD was really due to cholesterol removal from host cell membrane, cells pre-treated with the drug were subsequently incubated with WSC, an exogenous source of cholesterol which replenishes membrane cholesterol, washed with PBS and then exposed to *T. cruzi* TCTs. *T. cruzi* invasion into cholesterol-replenished cells was similar to that observed for non-treated control cells (Fig. 2A and C).

### Cholesterol depletion diminishes *T. cruzi* association with lysosomes during invasion into cardiomyocytes

In order to understand why cholesterol depletion was leading to a reduction in *T. cruzi* internalization, we investigate whether association between host cell lysosomes and *T. cruzi* was altered by MβCD incubation since this organelle is crucial for a successful parasite invasion [3], [4], [5], [25]. Untreated control cells, or cells treated with MβCD in different concentrations, were exposed to *T. cruzi* TCTs for 40 min., washed with PBS, fixed and submitted to the inside/outside parasite staining method, as well as labeled with LAMP-1 antibody for lysosomal membrane detection. Cholesterol removal from host cell membrane upon treatment with MβCD not only diminished host-cell invasion by the parasite (Fig. 2A and C), but also reduced the number of internalized parasites that had acquired lysosomal markers (Fig. 2B and C). Pre-treatment of cardiomyocytes with 10 mM MβCD caused a 60% reduction in the number of internalized parasites co-localizing with LAMP-1 (Fig. 2B). Moreover, increasing concentration of MβCD decreased both the number of internalized parasites (Fig. 2A), as well as the number of parasites co-localizing with the lysosomal marker in a dose-dependent manner (Fig. 2B). 15 mM of MβCD led to a reduction of 75% of lysosomal association with internalized trypomastigotes. Cells treated with HγCD, the inactive analog of MβCD, on the other hand, showed no statistically significant difference in *T. cruzi* invasion (Fig. 2A) or association of *T. cruzi* with host cell lysosomes when compared to control non-treated cells (Fig. 2B and C). Cholesterol replenishment not only re-established the ability of *T. cruzi* to invade host cells (Fig. 2A and C), but also elevated *T. cruzi* association with host cell lysosomes to values comparable with control non-treated cells (Fig. 2B and C).

### Cholesterol depletion reduces membrane raft labeling in cardiomyocytes

**Figure 3:**
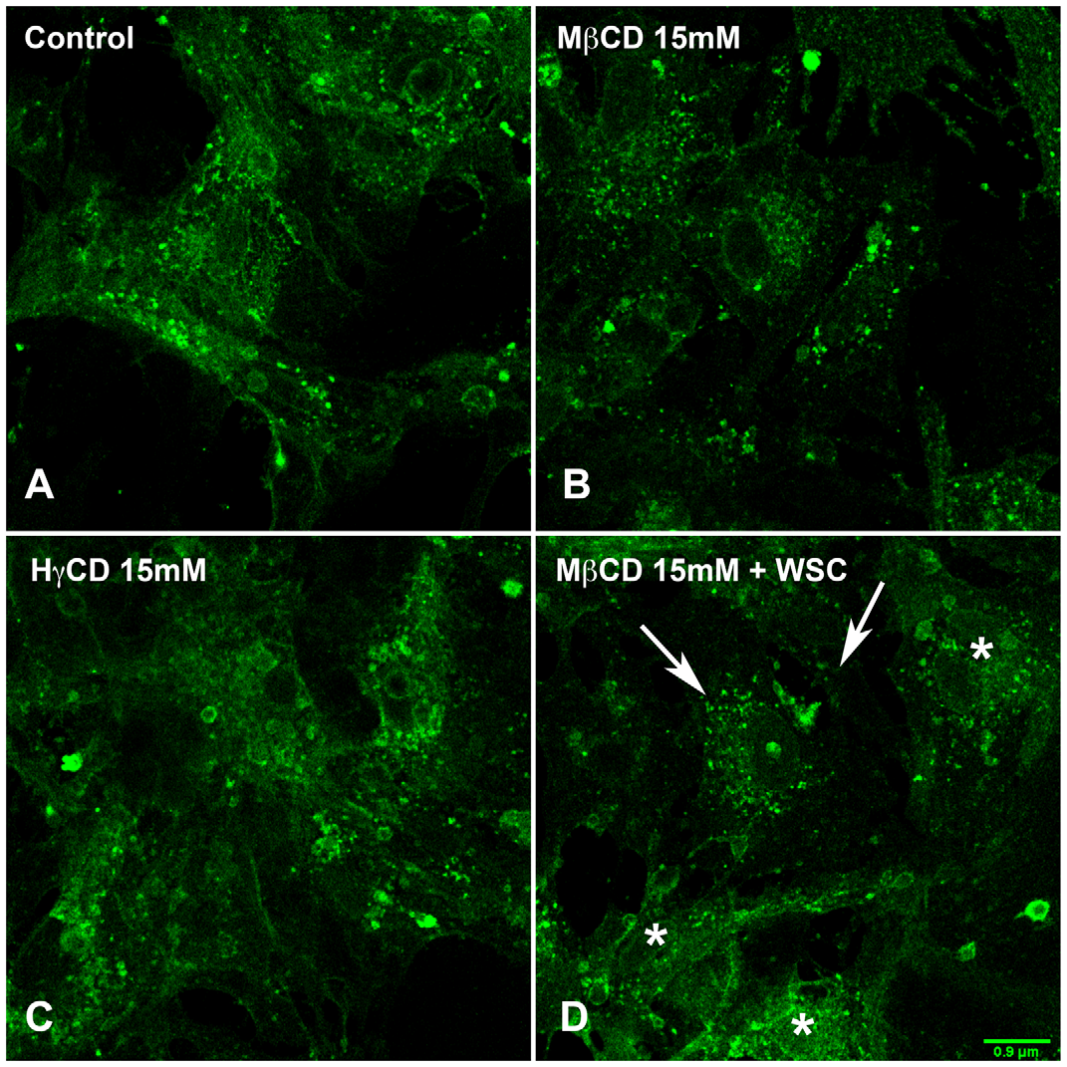
Treatment with MβCD leads to changes in membrane raft organization of cardiomyocytes. Confocal images of control (A) and cardiomyocytes pre-treated with 10 mM MβCD (B) or HγCD (C). Cells were washed, fixed and then labeled with CTXb-Alexa 488, which recognizes GM1, a raft marker. In comparison to control cells, which show a homogenous strong labeling for GM1, cholesterol-depleted cardiomyocytes reveals a more discrete labeling. Cells treated with HγCD show GM1 labeling similar to control cells whereas cholesterol-replenished cells (D) exhibit both patterns of cholesterol-depleted (arrows) as well as control (asterisks) GM1 labeling. Scale bar: 0.9 µm.

In order to evaluate how cholesterol depletion affected membrane raft organization, cardiomyocytes, which had their membrane cholesterol removed by treatment with 10 or 15 mM of MβCD, were fixed and labeled with subunit B of cholera toxin (CTXb). Subunit B of cholera toxin is a homopentamer that binds to GM1, a ganglioside that resides in membrane rafts, on the extracellular leaflet of plasma membrane [34], [35], [36]. Cells treated with 10 or 15 mM of HγCD or with 15 mM of MβCD, followed by cholesterol replenishment with 0.05 mM of WSC, were likewise stained. Cells with intact membrane cholesterol content show a more intense GM1 labeling, especially of larger lipid rafts (Fig. 3A) in comparison to cholesterol-depleted cells (Fig. 3B). In cardiomyocytes treated with HγCD (Fig. 3C), raft labeling was similar to control cells. In cholesterol-replenished cells (Fig. 3D), some cardiomyocytes retained a labeling pattern similar to that of cholesterol-depleted cells (arrows), while others showed a staining pattern more similar to untreated controls (asterisks). Therefore, MβCD treatment not only induced cholesterol sequestration from cell membranes, but also interfered with membrane raft organization in cardiomyocytes, which could not be totally recovered by cholesterol replenishment.

### Cholesterol depletion causes lysosomal exocytosis in cardiomyocytes

**Figure 4:**
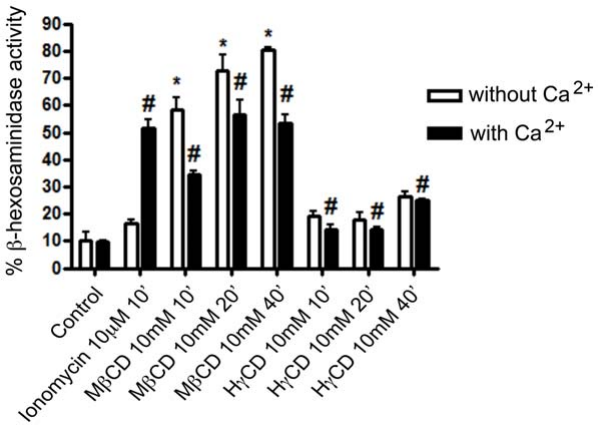
MβCD but not HγCD cell incubation leads to lysosomal exocytosis in cardiomyocytes. Cardiomyocytes were exposed to either 10 mM MβCD or HγCD for 10, 20 or 40 minutes at 37°C, in the absence (white bars) or presence (black bars) of calcium. Both extracellular media and lysates were collected and exposed to 4-methylumbelliferyl-N-acetyl-B-D-glucosaminide, the fluorescent substrate of beta-hexosaminidase, an enzyme resident within lysosomes. Results are shown as ratio between β-hexosaminidase activity in extracellular media and total β-hexosaminidase activity (extracellular media over extracellular media plus β- hexosaminidase cell lysate hexosaminidase activity). Cells treated with 10 µM Ionomycin (Calbiochem) for 10 minutes were used as lysosomal exocytosis positive control. Data are shown as mean of triplicates ±SD. Asteriks indicate statistically significant differences (p < 0.05,Student's t test) between control and treated cells.

Lysosomal fusion with the plasma membrane (lysosomal exocytosis), which occurs during *T. cruzi* entry into host cells, is an event regulated by calcium and proteins from the SNARE complex, in a mechanism similar to the fusion of synaptic vesicles with the pre-synaptic membrane [26], [37]. Depletion of cholesterol from the membrane has been shown to alter synaptic vesicle fusion with the plasma membrane leading to unregulated events of vesicle exocytosis [38]. In order to verify the behavior of lysosomal exocytosis in cholesterol-depleted cells, we performed a time-dependent assay in which the activity of beta-hexosaminidase, an enzyme resident within lysosomes, was measured in the extracellular media of cultured cells. Cardiomyocytes were incubated with 10 mM of either MβCD or HγCD and both the extracellular media and cell lysates of treated cells were incubated with a fluorescent substrate of beta-hexosaminidase. Non-treated and Ionomycin (an ionophore, which allows calcium influx into cells and induces lysosomal exocytosis [39]) treated cells were used as negative and positive controls, respectively. Experiments were performed in the presence or absence of calcium. Figure 4 shows that cardiomyocytes treatment with MβCD leads to lysosomal exocytosis events. As early as 10 minutes after the addition of the drug, in the presence of calcium, the rate of lysosomal exocytosis in cardiomyocytes was 3.5 times higher than control non-treated cells (Fig. 4). The levels were even higher the longer the incubation period with the drug. After 20 or 40 minutes of exposure with the drug the exocytosis level was about 5.5 times higher than control non-treated cells (Fig. 4). On the other hand, treatment with HγCD, the control drug, did not induce expressive exocytosis of lysosomal vesicles in cardiomyocytes (Fig. 4). To test whether these events were occurring spontaneously without calcium regulation, as was the case for synaptic vesicles, the same assay was performed in the absence of calcium and presence of the same concentration of magnesium. The extracellular calcium chelator, EGTA, did not inhibit lysosomal exocytosis induced by the incubation with MβCD. On the contrary, it seemed to slightly enhance exocytosis (Fig. 4).

**Figure 5:**
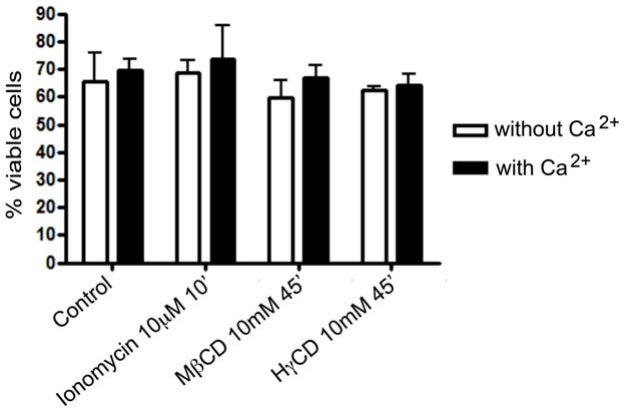
Lysosomal exocytosis events after cholesterol depletion are not due to cell death. After treatment with MβCD or HγCD, in the absence (white bars) or presence (black bars) of calcium, cardiomyocytes were trypsinized, collected and incubated with HFS solution, containing propidium iodide (PI). Cells that became inviable after drug treatment acquired PI labeling in their nuclei due to membrane permeability, and were counted as dead cells by flow cytometer. Data are shown as mean of triplicates ±SD.

To confirm that the high levels of beta-hexosaminidase observed in the extracellular media of MβCD treated cells were the result of triggered lysosomal exocytosis and not a consequence of cell injury upon treatment, a cell viability assay was performed. Cardiomyocytes treated or not with MβCD/HγCD were trypsinized and incubated with HFS solution, containing PI (propidium iodide), a nuclei dye impermeable to cell membranes. Negative controls (untreated cardiomyocytes) and positive controls (ionomycin treated cells) were also analyzed. Figure 5 shows that treatment with MβCD did not interfere with cell viability either in the presence or in the absence of calcium (Fig. 5). As expected, treatment with HγCD also did not lead to cell death.

### Cholesterol depletion changes lysosomal distribution in cardiomyocytes

**Figure 6:**
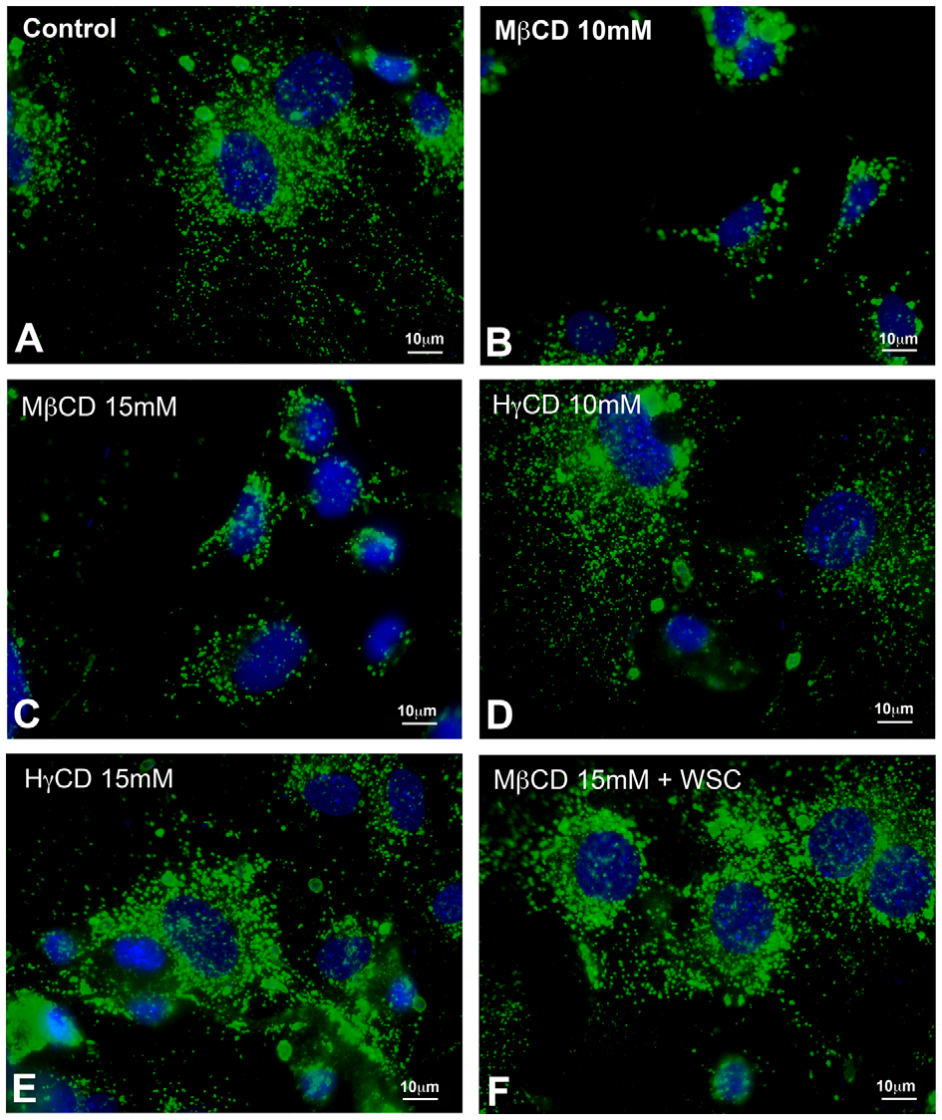
Cholesterol depletion leads to changes in lysosomal distribution within cells. Representative panels of lysosomal distribution in control cardiomyocyte (A) and cardiomyocytes pre-treated either with 10 mM (B) or 15 mM MβCD (C), 10 mM or (D) 15 mM HγCD (E), or 15 mM MβCD followed by 0.05 mM WSC (F). MβCD treated cardiomyocytes show significant changes in lysosomal dispersion in cell cytoplasm. After drug treatment, cells were washed and incubated with *T. cruzi* TCTs, M.O.I. of 50 parasites per cell for 40 minutes at 37°C. After cell invasion, cells were washed, fixed and immunostained for LAMP-1 (green) DAPI (blue) and analyzed under fluorescence microscope. In comparison to untreated cardiomyocytes (A), which exhibit homogenous and well distributed LAMP-1 labeling, MβCD treated cardiomyocytes, (B) and (C), show a heterogeneous LAMP-1 labeling with lysosomes localized predominantly near cell nuclei. On the other hand, HγCD treated cardiomyocytes and cholesterol-replenished cells, (E), (F) and (D), present lysosomal distribution similar to control cells. Scale bar: 10 µm.

**Figure 7:**
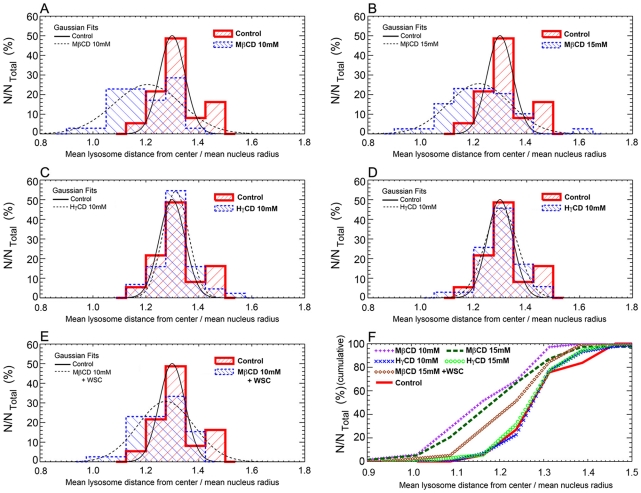
MβCD but not HγCD cell treatment leads to diminishment in lysosomal distribution near cell cortex. Quantitative analysis of lysosomal distribution, relative to cell nuclei, in 10 mM MβCD (A), 15 mM MβCD (B), 10 mM HγCD (C), 15 mM HγCD (D) or 15 mM MβCD followed by incubation with 0.05 mM WSC (E), treated cardiomyocytes in comparison to control non-treated cells. The mean distance between a lysosome and its respective cell center is represented by letter (D) and the mean lysosome distance (D) relative to the mean nucleus' radius (R) was defined as the ratio D/R. Ratio values closer to one indicate lysosomes are close to perinuclear region whereas the opposite indicate lysosomes are more frequent at cell borders. This ratio D/R was measured for several groups of lysosomes associated with each nucleus in the different treatments. The results of this analysis are distributions of D/R values associated to each drug treatment, and are represented as histograms. The histogram for each specific drug treatment is represented with a blue dashed line, which is superposed to the control (non-treated) lysosomal distribution, represented with a red solid thick line. Overlaid Gaussian fits to each distribution were added in order to highlight the main statistical trend of each diagram. The x-axis of each graph represents the ratio D/R whereas the y-axis represents the percentage of analyzed nuclei from each treatment normalized by total cell number. (F) Cumulative frequency of the histograms. Purple plus signs represent MβCD 10 mM treated cells, dark green rectangles represent MβCD 15 mM treated cells, blue crosses represent HγCD 10 mM treated cells, light green circles represent HγCD 15 mM treated cells, brown diamonds represent cells replenished with cholesterol after previous depletion and red continuous line represent control untreated cells. The Kolmogorov- Smirnov (KS) statistical test was performed to compare the cumulative frequency distributions. Statistically significant differences were obtained for MβCD 10 mM 0.02% (p = 0.0002) or MβCD 15 mM 0.03% (p = 0.0003) treated cells in relation to control, but not for HγCD 10 mM 79.49% (p = 0.7949), HγCD 15 mM 86.03% (p = 0.8603), or MβCD 15 mM + WSC 6.44% (p = 0.0644) treated cells in relation to control.

In order to understand the effect of exocytosis triggered by cholesterol removal from cell membranes on lysosomal distribution, images from cells submitted or not to treatment with MβCD, HγCD or MβCD + WSC and labeled with both DAPI (nuclei dye) and anti-LAMP-1 (lysosomal marker) were collected. Representative images of each condition are shown in figure 6. Qualitative analyses of the images revealed a more restricted distribution of lysosomes, closer to the cell nuclei, in cells treated with 10 mM or 15 mM MβCD (Fig. 6B and C), in comparison to control non-treated cells (Fig. 6A) or cells treated with HγCD, the MβCD inactive analog (Fig. 6D and E). Cholesterol replenishment after MβCD treatment seemed to revert the distribution of lysosomes to a pattern similar to the control non-treated cells (Fig. 6F). In order to precisely determine these differences, the same images were used to perform a quantitative assay of lysosomal dispersion (Fig. 7). First, for each isolated nucleus, the mean radius (R). was calculated. The next step was to select each lysosome associated with its respective nucleus and to measure the mean distance between a lysosome and cell center (D). Finally, the mean lysosome distance (D) relative to the mean nucleus' radius (R) was defined as the ratio D/R, where values closer to one indicate lysosomes are closer to perinuclear region whereas the opposite indicate lysosomes are more frequent at cell borders. This ratio D/R was measured for several groups of lysosomes associated with each nucleus in the different treatments. The results of this analysis are distributions of D/R values associated to each drug treatment, and are represented as histograms in Figure 7. Gaussian fits from control cells show that the majority of lysosomes are preferentially localized at ratio 1.3 from cell center whereas the peak of Gaussian fits from 10 mM MβCD treated cells show a ratio of 1.2 (Fig. 7A). The same pattern is seen upon treatment with higher concentrations of the drug (Fig. 7B). Moreover, lysosomes at higher ratios, in other words more distant from the cell nuclei, are mostly found in control non-treated cells, with none or only a few found in MβCD treated cells (Fig. 7A and B). On the other hand, no difference in lysosomal distribution was observed when cells were treated with 10 or 15 mM of HγCD as compared to control non-treated cells (Fig. 7C and 7D). Finally, cholesterol replenishment after MβCD treatment was able to, at least in part, revert lysosomal dispersion to a pattern more similar to control cells (Fig. 7E). Cumulative frequencies of lysosomes (Fig. 7F) from the histograms of figures 7A to E were plotted and analyzed using Kolmogorov-Smirnov statistical test. Statistically significant differences were only observed between 10 or 15 mM of MβCD treated and control non-treated cells. 10 and 15 mM HγCD or 15 mM of MβCD + WSC treated cells presented cumulative frequencies similar to control non-treated group. In order to prove that this rearrangement in lysosome distribution was not a consequence of cell surface area decrease upon cholesterol removal from plasma membrane, cells were treated or not with MβCD or its inactive analog, HγCD, as well as MβCD + WSC, and labeled with the plasma membrane stain, CellMask (Invitrogen). Images were collected and cell surface area measured using the ImageJ software. No difference in cell surface area was found among the distinct groups (Supplementary Fig. 1).

## Discussion

Two different groups have previously demonstrated the participation of cholesterol and membrane rafts as “hot spots” for *T. cruzi* entry into host cells [22], [23]. In 2007 Fernandes and co-workers showed that cholesterol and membrane rafts participate in the internalization of metacyclic trypomastigotes and extracellular amastigotes from two different strains, CL and G, into non-professional phagocytic cells. In this same year, Barrias and co-workers verified the participation of membrane rafts in the internalization of *T. cruzi* TCTs in phagocytic cells (murine peritoneum macrophages). However, in both studies it was not clear how cholesterol and/or rafts participated in the process of parasite entry into host cells. In the present work we show the participation of cholesterol in *T. cruzi* TCT entry into non-professional phagocytic cells and, most importantly, the mechanism by which plasma membrane cholesterol interferes with parasite invasion into non-professional phagocytic cells.

In order to investigate the participation of cholesterol in *T. cruzi* TCT invasion of non-professional phagocytic cells, we pre-treated cells with different concentrations of MβCD followed by invasion assays with *T. cruzi*. Cyclodextrins, like MβCD, are oligosaccharides constituted by glucopyranose units that are linked by α-(1-4) bonds [40]. These compounds are broadly used as liposoluble drug carriers since they are soluble in water and have a hydrophobic core in which non-soluble substances are transported [41]. β-cyclodextrins, especially MβCD, present a higher affinity for cholesterol as compared to the α and γ cyclodextrins, [42]. Labeling MβCD treated cardiomyocytes with Filipin III (a fluorophore with high affinity for cholesterol) confirmed the ability of the drug to remove cholesterol from cell membranes.

As previously shown for the metacyclic trypomastigotes and extracellular amastigotes forms of *T. cruzi*, we showed that a reduction in host cell surface cholesterol decreases the rate of invasion of non-professional phagocytic cells by *T. cruzi* TCTs, even though these forms of the parasite present different surface molecules and consequently stimulate cells by distinct mechanisms [43]. This effect on TCT host cell entry was indeed due to cholesterol removal from host cell plasma membrane, since invasion assays with cells previously treated with HγCD, an inactive analog of MβCD which presents very low affinity for cholesterol, did not change the parasite invasion profile in comparison to control untreated cells. Also, host cell viability was not compromised after treatment as shown by a cell viability assay, confirming that the observed reduction in host cell invasion levels was not a result of cell loss due to drug treatment. Moreover, cholesterol replenishment after cell treatment with MβCD re-established invasion to control levels. Together, these results undoubtedly show that cholesterol is also important for *T. cruzi* TCT entry into cardiomyocytes and that this model can be used to investigate the role of cholesterol in this process. We found that the low invasion rate of *T. cruzi* into cholesterol-depleted cells was accompanied by a diminishment in lysosome recruitment, which is required for the formation of the parasitophorous vacuole. Lysosome recruitment and fusion has been shown to be essential not only for inducing parasite internalization, but also for holding *T. cruzi* inside host cells [3], [44]. Fusion of lysosomes with host cell plasma membrane induced by *T. cruzi* leads to a compensatory endocytic pathway that drives parasites into cells [44]. Parasites are known to tightly interact with parasitophorous vacuolar membrane, probably through lysosomal integral membrane proteins such as LAMP [45], [46]. Membrane fusion events, such as synaptic vesicle and lysosomal exocytosis as well as other types of vesicle secretion, are regulated by calcium and occur through a mechanism dependent on proteins from the SNARE complex [26], [47], [48]. It is well known from microscopy studies that these proteins concentrate in submicrometre-sized, cholesterol-dependent clusters, such as membrane rafts, at which sites vesicles fuse [27], [28]. Since membrane rafts are cholesterol-enriched microdomains (about 50% of total cellular cholesterol) located in cell plasma membrane [42] and cholesterol removal from cell membranes induces changes in raft organization and function [49], [50], [51], it is possible that the effect of cholesterol removal on *T. cruzi* entry was a consequence of the disruption of these microdomains. GM1 labeling, a known raft marker, has demonstrated that MβCD treatment of cardiomyocytes leads to changes in raft organization in these cells, suggesting a role not only for cholesterol but also for membrane raft microdomains in TCT's invasion of non-professional phagocytic cells. Raft disorganization, on the other hand, could alter membrane fusion events, by changing SNARE proteins distribution and/or function, disturbing the exocytic events regulated by these proteins. In fact, cholesterol removal led to massive non-regulated lysosomal exocytosis events, which occurred in the absence of calcium, suggesting that disruption of raft organization de-regulates lysosomal exocytosis. Corroborating this idea, it has been demonstrated for neuronal exocytosis that SNARE localization in rafts work as negative regulators of secretion and reducing SNAP 23 partitioning to raft sites enhanced vesicle exocytosis [52]. Interestingly, SNAP 23 is one of the SNARE complex proteins involved in lysosomal fusion events [26]. Similar exocytic events, triggered by treatment with MβCD, have already been demonstrated in other animal models. Zamir and Charlton (2006) [53], analyzing neuromuscular junctions in crayfish, realized that treatment with 10 mM MβCD induced a 5-fold increase in the rate of spontaneous miniature excitatory post synaptic potentials (mEPSPs), as a consequence of unregulated, calcium independent, synaptic vesicle fusion events. Other authors have also shown changes in vesicle secretion upon cholesterol removal from plasma membrane [38], [54], [55], [56]. Recently, Chen and co-workers studying cells derived from a mouse model of Niemann-Pick disease (a disorder characterized by a massive accumulation of lipids, including cholesterol, in the endosomal/lysosomal system) have shown that treatment with hydroxypropyl-β-cyclodextrin (HPβ-CD), a cyclodextrin similar to MβCD, leads to lysosomal exocytosis, as early as 15 minutes post exposure to the drug [57]. This result corroborates our data since cell incubation with MβCD also led to lysosomal exocytosis at early time points. However, contrary to what was observed by these authors, lysosomal exocytosis triggered by incubation with MβCD in cardiomyocytes is independent of extracellular calcium. It is still possible though that intracellular calcium is responsible for these exocytic events. Another possibility, since HPβ-CD and MβCD differ in their efficiency of extracting cell membrane cholesterol, is that the effect of these drugs on exocytosis might be different [42]. In fact, it has been shown that the effect of MβCD on spontaneous release of synaptic vesicles, generating mEPSPs in neuromuscular junctions, occurs in the absence of intracellular and extracellular calcium [53].

Finally, since our data shows that lysosomal exocytosis happened in the early stages of MβCD treatment, one could assume that a significant reduction in lysosomal reservoir occurred during the period of drug incubation. Quantitative analysis of lysosomal distribution in cells before and after cholesterol depletion showed that control cells have their lysosomal pool well distributed throughout the cell cytosol, with vesicles around the perinuclear and cell cortex area (Fig. 6A, qualitative image). However, when cholesterol is sequestered by MβCD, only the lysosomes near the perinuclear area remain (compare Fig. 6A and B), without a change in cell surface area upon treatment (Figure S1). Based on these results it is plausible to assume that cholesterol depletion evokes exocytosis of docked lysosomes localized near the cell cortex. Taken together, these data suggest the existence of two independent lysosomal pools (one near the cell surface and another in the perinuclear area), which might be differentially regulated. The docked lysosomes near the cell surface would then represent the pool triggered by *T. cruzi* and therefore involved in the exocytic events that initiate its internalization process. Without enough lysosomes available at the cell surface for fusion and formation of the parasitophorous vacuole, *T. cruzi* entry is compromised.

We cannot discard however that reduction in membrane cholesterol content and its consequent raft disorganization may also affect intracellular signaling pathways [58], [59], [60]. Therefore, receptors present in membrane rafts, which might be important for recognition and signal transduction during *T. cruzi* interaction and internalization into host cells, may have their functions attenuated or compromised and consequently affect parasite invasion rates. In this sense the diminishment in *T. cruzi* association with lysosomes observed upon treatment with MβCD could be also partly due to the compromised function of these receptors. Studies are being carried out to evaluate these possibilities.

## Supporting Information

Figure S1 **Cholesterol depletion does not change the cell area of cardiomyocytes.** (A) Representative figure of control, 15 mM MβCD, 15 mM HγCD and cholesterol-replenished cells labeled with CellMask Orange plasma membrane stain. Briefly, cardiomyocytes were treated with the cyclodextrins, washed and incubated with a 5 µg/mL solution of CellMask in fresh medium for 5 minutes, at 37°C. After that period, cells were fixed for 10 minutes, at 37°C, washed and mounted with antifade medium and analyzed immediately in a confocal microscope. (B) Histograms showing distributions of cell areas for different treatments (MβCD 15 mM- black squares line; HγCD 15 mM- blue square-traces line and MβCD 15 mM followed by 0.05 mM WSC- green rectangles line in comparison to control cells (red continuous line). In the cumulative frequence distributions, statistical KS test results shows that control vs MβCD 15 mM is 14.8% (p = 0.148); control vs HγCD 15 mM is 14.3% (p = 0.143) and control vs WSC treated cells is 55.5% (p = 0.555). Altogether this statistical analysis shows that there are no differences between areas in control or cyclodextrin treated cells.
(TIF)
