# Supplementary figures and images for: Membrane Cholesterol Regulates Lysosome-Plasma Membrane Fusion Events and Modulates Trypanosoma cruzi Invasion of Host Cells
Source: PLoS Negl Trop Dis. 2012 Mar 27;6(3):e1583. doi: 10.1371/journal.pntd.0001583 (PMC3313932; doi:10.1371/journal.pntd.0001583)

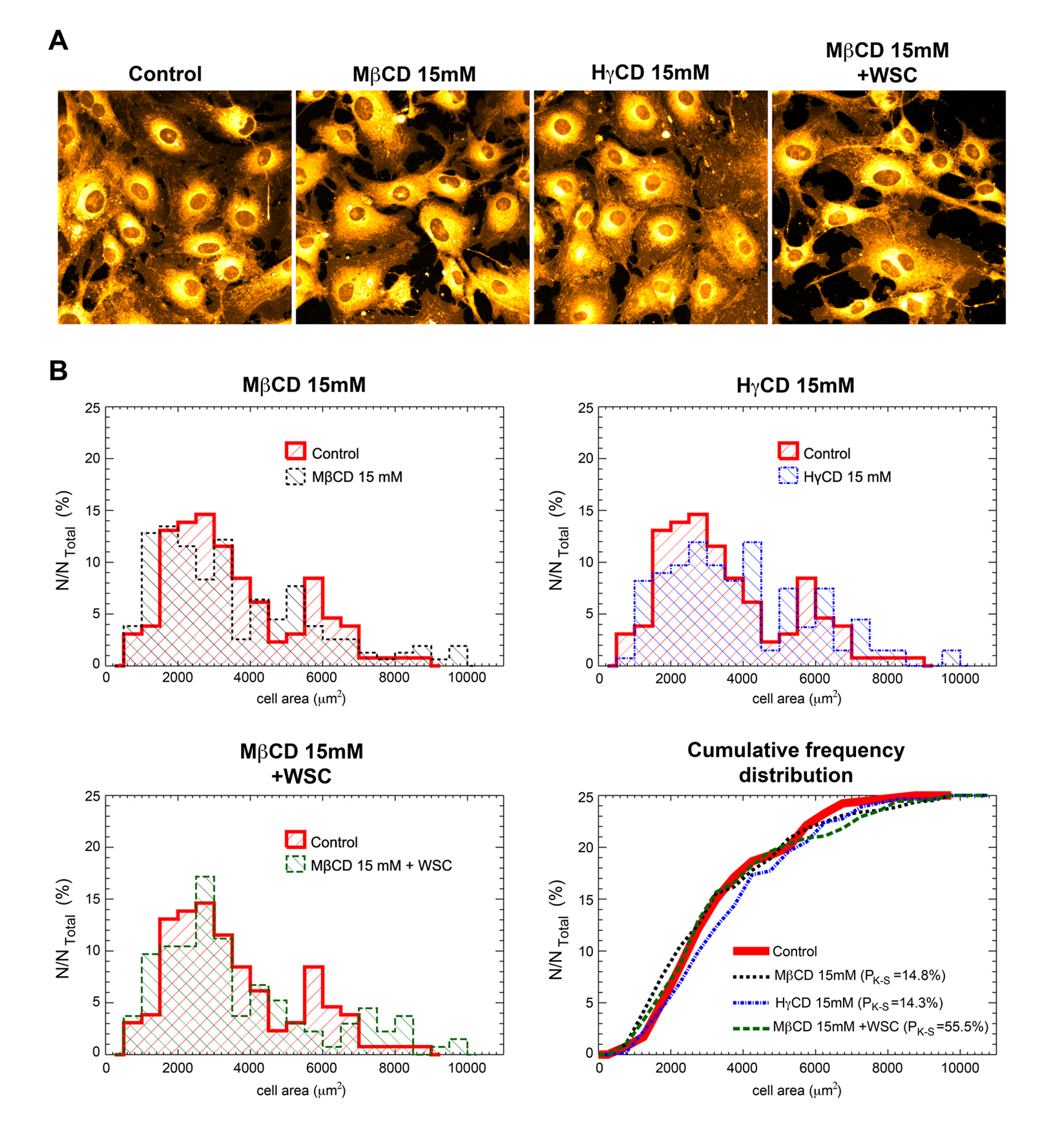

Supplement: Figure S1 — Cholesterol depletion does not change the cell area of cardiomyocytes. (A) Representative figure of control, 15 mM MβCD, 15 mM HγCD and cholesterol-replenished cells labeled with CellMask Orange plasma membrane stain. Briefly, cardiomyocytes were treated with the cyclodextrins, washed and incubated with a 5 µg/mL solution of CellMask in fresh medium for 5 minutes, at 37°C. After that period, cells were fixed for 10 minutes, at 37°C, washed and mounted with antifade medium and analyzed immediately in a confocal microscope. (B) Histograms showing distributions of cell areas for different treatments (MβCD 15 mM- black squares line; HγCD 15 mM- blue square-traces line and MβCD 15 mM followed by 0.05 mM WSC- green rectangles line in comparison to control cells (red continuous line). In the cumulative frequence distributions, statistical KS test results shows that control vs MβCD 15 mM is 14.8% (p = 0.148); control vs HγCD 15 mM is 14.3% (p = 0.143) and control vs WSC treated cells is 55.5% (p = 0.555). Altogether this statistical analysis shows that there are no differences between areas in control or cyclodextrin treated cells. (TIF) [file pntd.0001583.s001.tif]
